# Single-Ion Counting with an Ultra-Thin-Membrane Silicon Carbide Sensor

**DOI:** 10.3390/ma16247692

**Published:** 2023-12-18

**Authors:** Enrico Sangregorio, Lucia Calcagno, Elisabetta Medina, Andreo Crnjac, Milko Jakšic, Anna Vignati, Francesco Romano, Giuliana Milluzzo, Marzio De Napoli, Massimo Camarda

**Affiliations:** 1 Department of Physics and Astronomy “Ettore Majorana”, University of Catania (Italy), Via Santa Sofia 64, 95123 Catania, Italy; enrico.sangregorio@phd.unict.it; 2 STLab srl, Via Anapo 53, 95126 Catania, Italy; elisabetta.medina@unito.it (E.M.); massimo.camarda@stlab.eu (M.C.); 3 Physics Department, Università degli Studi di Torino, Via Pietro Giuria 1, 10125 Turin, Italy; anna.vignati@unito.it; 4 INFN—National Institute for Nuclear Physics, Turin Division, Via Pietro Giuria 1, 10125 Turin, Italy; 5 Division of Experimental Physics, Ruđer Bošković Institute, 10000 Zagreb, Croatia; jaksic@irb.hr; 6 INFN—National Institute for Nuclear Physics, Catania Division, Via S. Sofia 64, 95123 Catania, Italy; francesco.romano@ct.infn.it (F.R.); giuliana.milluzzo@ct.infn.it (G.M.); marzio.denapoli@ct.infn.it (M.D.N.); 7 SenSiC GmbH, DeliveryLAB, 5234 Villigen, Switzerland

**Keywords:** silicon carbide, membrane sensor, deterministic ion implantation, counting efficiency, spatial resolution

## Abstract

In recent times, ion implantation has received increasing interest for novel applications related to deterministic material doping on the nanoscale, primarily for the fabrication of solid-state quantum devices. For such applications, precise information concerning the number of implanted ions and their final position within the implanted sample is crucial. In this work, we present an innovative method for the detection of single ions of MeV energy by using a sub-micrometer ultra-thin silicon carbide sensor operated as an in-beam counter of transmitted ions. The SiC sensor signals, when compared to a Passivated Implanted Planar Silicon detector signal, exhibited a 96.5% ion-detection confidence, demonstrating that the membrane sensors can be utilized for high-fidelity ion counting. Furthermore, we assessed the angular straggling of transmitted ions due to the interaction with the SiC sensor, employing the scanning knife-edge method of a focused ion microbeam. The lateral dimension of the ion beam with and without the membrane sensor was compared to the SRIM calculations. The results were used to discuss the potential of such experimental geometry in deterministic ion-implantation schemes as well as other applications.

## 1. Introduction

Ion implantation has been widely applied in the semiconductor industry, as introducing dopants is an easy and fast way to engineer the electrical and optical properties of semiconductors. Over the last decades, deterministic single-ion implantation has attracted wide interest in the semiconductor field because of its application in solid-state quantum technology across various material systems, including silicon and diamond. Some non-exhaustive examples of Si-based single-dopant devices include donors coupled to quantum dots [1] for charge [2], electron [3,4], and nuclear spin [5,6] qubits (quantum bits). Alternatively, single-color centers in diamond substrates, including nitrogen-vacancy (NV) centers [7] and coupling silicon-vacancy (SiV) centers [7,8], are studied as quantum electrodynamics (QED) devices based on diamond technology. Motivated by the proposed quantum applications, the demand for deterministically placing single dopants into nanostructured devices has prompted the development of various techniques related to silicon and diamond material systems [9,10]. Single-ion implantation is achieved through accurate control over the ion’s final position and the number of implanted ions. Single-atom lithographic techniques based on scanning probes have successfully achieved the positioning of single dopants with nanometer-scale precision [11,12,13]. However, this technique is currently limited to a small number of species and is relatively slow. In contrast, direct ion implantation offers less precision in terms of dopant atom positioning but offers more flexibility in the choice of ion species, potentially allowing for faster and more scalable processes [9,14]. The primary challenges in direct ion-implantation methods lie in accurately counting individual ions as they approach the substrate and precisely predicting their final position within the sample. Several techniques have been developed to monitor the number of ions reaching the sample during single-ion-implantation processes. One of the most commonly used methods involves the detection of secondary electrons emitted upon the impact of ions on the sample [15,16]. This approach requires the presence of a secondary electron detector and can be applied to nearly all samples (provided that the ion energy is sufficiently high to yield a detectable secondary electron emission). Alternatively, other frequently used techniques exploit integrated structures within the implanted sample to generate detectable signals during the process. Integrated structures such as PiN diodes, which utilize electron–hole pairs generated by ion–matter interactions, can produce a detectable signal upon free-charge capture [17,18]. Additionally, integrated field-effect transistors (FETs) are employed, where ion implantation modulates the drain current [18,19]. However, these methods are exclusively applicable to samples featuring PiN or FET structures, thereby limiting their utility in various areas.

In the present work, we present an innovative single-ion detection method using an independent silicon carbide sensor to be placed ahead of the sample to be implanted. The sensor is a sub-micrometer SiC membrane realized through a state-of-the-art, doping-selective electrochemical etching process [20,21]. The described sensor geometry was employed here for detecting ion beams in the MeV energy range. In this setup, the ions lose only a portion of their energy (ΔE) in the device and are transmitted further without significant influence on the impact trajectory and total energy (E) of the ions. The electron/hole pairs generated through the ion–sensor interaction are amplified and collected, resulting in a distinct signal corresponding to each ion passing through the SiC membrane. The results were compared with measurements obtained using a reference silicon detector. Moreover, a study of the alteration in the ion beam after crossing the membrane was conducted, and the lateral ion straggling, a crucial parameter for single-ion-implantation applications, was calculated and compared with simulations.

## 2. Materials and Methods

The device tested is an advanced silicon carbide ultra-thin radiation sensor engineered as a free-standing membrane with a parallel-plate electrode configuration. This device is a semiconductor Schottky barrier diode consisting of an ultra-thin n^−^ silicon carbide active layer characterized by a low doping concentration of 10^14^ cm^−3^ on top of an inert n^+^ highly doped silicon carbide substrate approximately 370 µm thick with a doping concentration of 10^18^ cm^−3^. The fabrication of the free-standing membrane at the core of the sensor was accomplished through a state-of-the-art, doping-selective electrochemical etching (ECE) technique, which allowed for precision material removal down to sub-micrometer thickness levels. In more detail, the electrochemical etching of silicon carbide in hydrogen fluoride (HF)-based solutions consists of two steps: the first step is the oxidation of SiC driven by holes, and the second step is the dissolution of the formed SiO_2−x_ in HF [22]. In the case of the highly doped 370 µm substrate, holes were generated by tunneling effects. In contrast, for the low-doped n-type SiC epitaxial layer, tunneling was negligible. Therefore, the thin epitaxial layer acted as a stopping layer for the etching process, hence resulting in the formation of the free-standing membrane [20]. The total sensor area (5 × 5 mm^2^) was divided into four independent pads, and the ECE process was carried out within a 2 mm diameter circular region in the central area of the device. In this study, only one of the four pads was connected to the data acquisition system and analyzed.

**Figure 1:**
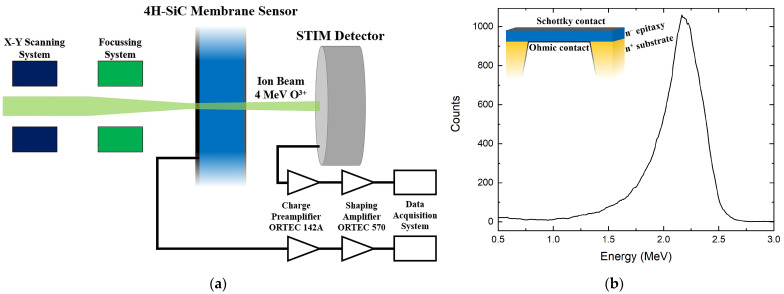
(**a**) Schematic of the experimental setup for the calculation of the sensor thickness and counting measurements; (**b**) energy spectrum obtained by the STIM detector used for the determination of the SiC sensor thickness. In the insert, a schematic cross-section of the SiC sensor is shown.

The metal contact, needed for both the ECE process and for the subsequent sensor operation, was established by depositing a 30 nm aluminum layer on the front surface of the device to create a Schottky contact. In contrast, the back contact was applied after the ECE process and involved a 100 nm aluminum layer. The metal depositions were conducted using an electron-beam (E-beam) evaporation system. A schematic structure of the SiC membrane sensor is presented in the inset of Figure 1b.

The main characterization of the sensor was performed by exposing the device to accelerated ions of different masses and energies in the MeV range using the ion beam facility of the Ruđer Bošković Institute [23]. Techniques based on the interaction of MeV ion beams with materials offer a powerful analytical framework for semiconductor detector characterization [24,25]. The device was mounted in a vacuum irradiation chamber attached to the 6 MV Tandem Van de Graaff electrostatic accelerator. The accelerator is equipped with a sputtering ion source used for the production of a wide range of ion species, from light ions such as H or Li to very heavy ions (up to Au). Ions are accelerated and transmitted through a range of ion beam optics elements downstream to the experimental end station, where the samples are positioned. In our setup, an ion microprobe end station was employed, allowing the focusing of ion beams to a micrometer-sized spot and enabling the scanning of the beam across the sample surface. These experimental conditions were utilized to acquire spatially resolved information about the ion–sample interaction as determined by the beam spot size. This setup was crucial for testing the device for single-ion detection, as the ion beam current could be reduced to ~ Hz rates and positioned in different spatial regions of interest of our device.

In our experimental scenarios, the ion beam was transmitted through the SiC membrane portion of the sensor, leaving only a portion of the energy inside, and was stopped 6 cm downstream on the in-beam-positioned Si detector, which was a Passivated Implanted Planar Silicon (PIPS) detector with very thin top dead layer. The PIPS detector will be referred to as a Scanning Transmission Ion Microscopy (STIM) detector (Canberra Semiconductor, Olen, Belgium), as it was used to detect transmitted ions. Both devices were connected to the same low-noise signal-processing chain, which was based on a charge-sensitive preamplifier (ORTEC 142A) and a shaping amplifier (ORTEC 570), both provided by ORTEC, Oak Ridge, TN, USA. This geometry enabled independent detection of ions by the device under test (the SiC membrane) and a well-characterized solid-state Si detector (the STIM detector). When an ion interacts with a semiconductor detector, it produces electron–hole pairs that are collected by the electric field applied through the electrodes. This technique is often referred to as the ion beam induced charge (IBIC) technique [24]. Signals collected from the sensor electrode can be used to quantify different parameters, such as the deposited energy, transient collection behavior, timing properties, and so on. When combined with a scanning microbeam setup, the IBIC technique can be seen as a 3D-like microscopic technique for the investigation of charge transport properties in semiconductor detectors.

A 4 MeV O^3+^ ion beam was employed to precisely determine the thickness of the silicon carbide membrane. A schematic representation of the experimental setup used during this investigation is presented in Figure 1a. The incident ions that passed through the SiC sensor deposited a portion of their energy (ΔE, approximately 45%) within the free-standing membrane sensor. Subsequently, these ions were collected by the STIM detector, and the acquired signal was represented as a count-versus-energy plot (Figure 1b). After subtracting the energy deposited in the STIM detector, which is represented by the peak position in Figure 1b, from the initial ion beam energy, the energy deposited in the SiC membrane sensor (ΔE) was determined. Furthermore, the full-width half maximum of the peak in Figure 1b was used to calculate the uncertainty in the membrane thickness. The Stopping and Range of Ions in Matter (SRIM) Monte Carlo simulation tool [26] was used to estimate the energy loss ΔE/Δx (eV/nm) of the beam in the sensor, enabling the calculation of the membrane sensor thickness T_SiC_. Using this method, a total sensor thickness of T_SiC_ = 727.3 ± 57.6 nm was calculated. Considering the thickness of the aluminum electrodes, the membrane active layer thickness resulted in about 597 nm. The relatively high 8% error associated with this measurement can be primarily attributed to the surface roughness of the membrane resulting from the doping-selective ECE process used for the formation of the SiC membrane [20,21]. The ion-counting fidelity of the SiC membrane was determined using the same oxygen beam and experimental setup while employing the IBIC technique.

The electron–hole pairs generated as a result of energy deposition during ion–membrane interactions were collected, and 2D-IBIC maps were generated using the homemade software SPECTOR v2.0 [27]. During the acquisition of IBIC signals, a reverse bias of −5 V was applied to the SiC Schottky diode. After this first interaction, the ions had enough energy to reach the STIM detector, thereby allowing for a simultaneous generation of a second IBIC map corresponding to transmitted ions. A comparison between the two acquired maps was performed to determine the number of recorded events in the two devices while assuming a 100% collection efficiency in the STIM detector. This comparison enabled the evaluation of the single-ion detection efficiency of the SiC membrane sensor.

The decrease in ion energy was not the sole effect of the interaction between the ion beam and the SiC membrane sensor. As the ions collide with the atomic electrons of the solid sensor, the trajectory angle of the ions in the material can be altered [25]. This phenomenon, commonly denoted as “ion lateral straggling”, increases the uncertainty in the final position of ions within the implanted sample. In our experiment, we quantified ion lateral straggling resulting from the interaction between ions and the SiC sensor by using a finely machined metal grid with a defined pitch dimension. The grid was positioned between the membrane and the STIM detector, allowing the scanning transmitted ion beam to form a projection image of the grid. Using this experimental setup, a 10 MeV C^4+^ ion beam was scanned across the grid to acquire 2D-IBIC maps both with and without the presence of the membrane. This enabled the determination of the beam spot dimension in the two cases using the knife-edge analysis technique based on the grid projection.

## 3. Results

### 3.1. Counting Measurement

**Figure 2:**
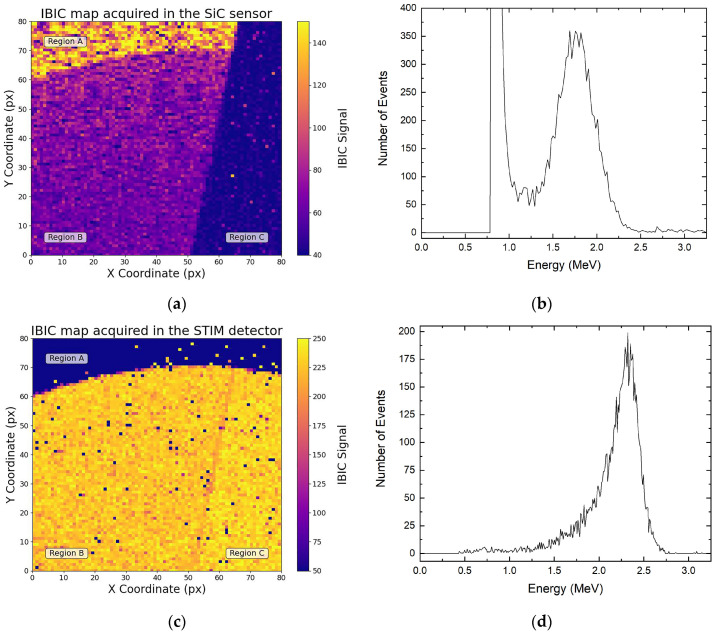
(**a**) IBIC map acquired by the SiC sensor and the corresponding energy spectrum (**b**); (**c**) IBIC map acquired by the STIM detector and its corresponding energy spectrum (**d**). Both maps were acquired simultaneously using a 4 MeV O^3+^ ion beam.

Figure 2a shows an IBIC map obtained with a 4 MeV O^3+^ beam on the SiC membrane. Since IBIC signals are proportional to the amount of energy deposited by ions, three different regions are visible on the map. Region A corresponds to an area where the 370 µm bulk beneath the membrane remained intact after the etching process. In this region, the elevated IBIC signal can be explained by the contribution of two types of free charges to the overall signal: charges generated in the membrane that were driven to the sensor electrodes by the electric field (drift current) and charges produced in a shallow region of the bulk below that reached the electrodes through diffusion processes (diffusion current). In region B, the membrane signal was acquired. In this region, only the charges generated in the epitaxial membrane were collected on the sensor electrodes, and a lower IBIC signal was generated. Finally, region C represents a non-bonded sensor pad, therefore this region is dominated by background noise. Figure 2c shows the IBIC map acquired by the STIM detector simultaneously with the events mapped in Figure 2a. Here, region A exhibits no STIM signal due to the thick silicon carbide substrate, in which the ions are fully absorbed and not transmitted to the PIPS detector. Region B and part of region C represent the membrane section of the SiC sensor, which shows a STIM signal, as the ion of the beam had enough energy to cross the membrane and reach the silicon reference detector.

To assess the SiC sensor’s capability to detect individual ions, a comparative analysis was performed between the counts recorded independently in the two detection systems. Assuming a 100% detection efficiency for the STIM detector, the relative SiC detection efficiency was determined accordingly. Identical regions in both maps were selected, and the number of event histograms (corresponding only to the ions recorded within those regions of interest) were extracted both for the SiC sensor (Figure 2b) and the STIM detector (Figure 2d).

The count plot in Figure 2b shows a high-count, low-energy (0.8 MeV) peak attributed to electronic noise. In the process of quantifying the number of ions detected by the membrane sensor, these noise events were removed from the total count, resulting in the exclusive contribution of the 1.7 MeV signal due to the overall membrane sensor. The extended low-energy tail observed in the histogram of transmitted ion energies (Figure 2d) can be attributed to the aforementioned energy-straggling effects, leading to the generation of an asymmetric 2.3 MeV peak. The difference in the peak position in Figure 2b,d derived from the different energy that the 4 MeV ions deposited in the two sensors. This procedure was repeated for various areas within region B, and the comparison of events recorded simultaneously by the two detection systems resulted in a 96.5 ± 0.9% ion-detection efficiency for the SiC sensor. The approximately 3% difference in the recorded events within the membrane sensor can be related to the complexities involved in subtracting electronic noise from the signal, which was primarily due to the proximity of the two peaks and the asymmetry of the membrane signal. While further improvements can be made by reducing background noise, this result underscores the excellent charge-collection efficiency of the tested ultra-thin membrane, highlighting its strong potential for high-fidelity ion-counting applications.

### 3.2. Lateral Straggling

In single-ion-implantation applications, careful control is applied over the implantation process to guarantee the precise localization of each ion within the target material. However, the occurrence of ion straggling resulting from the interactions between the ion beam and the independent ion sensor has the potential to compromise the determinism of the implantation procedure. This phenomenon introduces uncertainty regarding the final position of the implanted ions. Furthermore, the lateral straggling discussed earlier should be considered in conjunction with other non-improvable phenomena such as lateral straggling arising from ion–sample interaction and diffusion processes occurring during post-implantation annealing, which is essential for the activation of implanted species.

**Figure 3:**
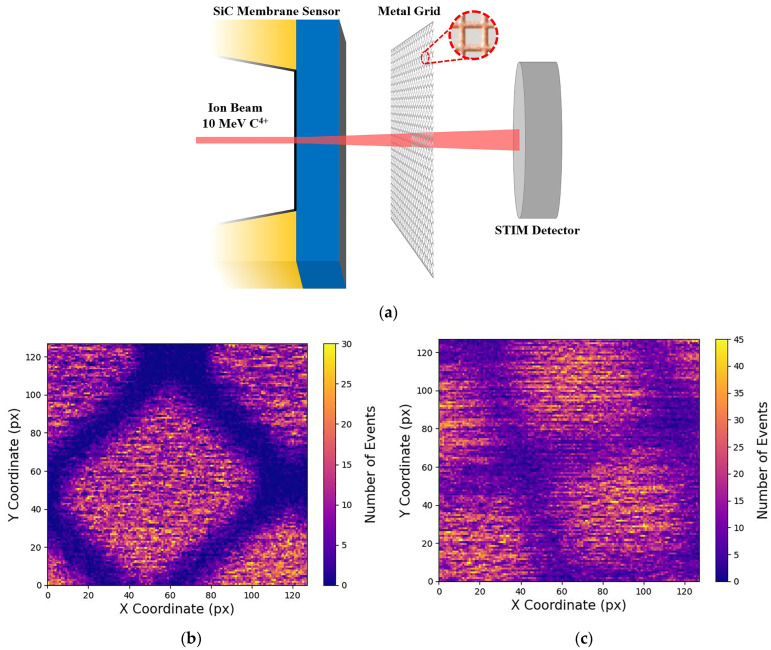
(**a**) Schematic representation of the experimental setup used for the lateral straggling determination; (**b**) STIM map acquired without the SiC membrane sensor; (**c**) STIM map acquired with ions passing through the SiC membrane sensor. Both maps were acquired using a 10 MeV C^4+^ ion beam.

The ion lateral straggling was calculated by measuring the spatial profile of the ion beam before and after interaction with the silicon carbide free-standing membrane sensor. To achieve this, an electroformed metal grid with a well-known pitch dimension of 25.4 µm was mounted inside the experimental chamber between the SiC membrane and the downstream STIM detector (Figure 3a). The function of the grid was to act as an obstacle for the ions as they were transmitted toward the downstream detector, resulting in the generation of IBIC maps with the projected image of the grid formed by ions passing through the open regions of the grid. This allowed us to determine the ion beam spot size at the plane of the grid.

With this experimental setup, a 10 MeV C^4+^ ion microbeam was focused on the PIPS detector, and STIM data were acquired under two different configurations. In the first configuration, the carbon ion beam traversed the grid and reached the PIPS detector without any interaction with the silicon carbide free-standing membrane. In this case, the grid edges of the resulting STIM signal exhibited a high-definition level due to the unaltered ion beam’s convergence (Figure 3b). In the second scenario, the SiC membrane was mounted before the metal grid, causing the ion beam to interact with the SiC membrane before encountering the grid. As previously mentioned, this interaction between the SiC sensor and the carbon ions introduced straggling effects. Consequently, the divergence of the ion beam led to a lower edge definition of the grid in the final STIM map (Figure 3c).

**Figure 4:**
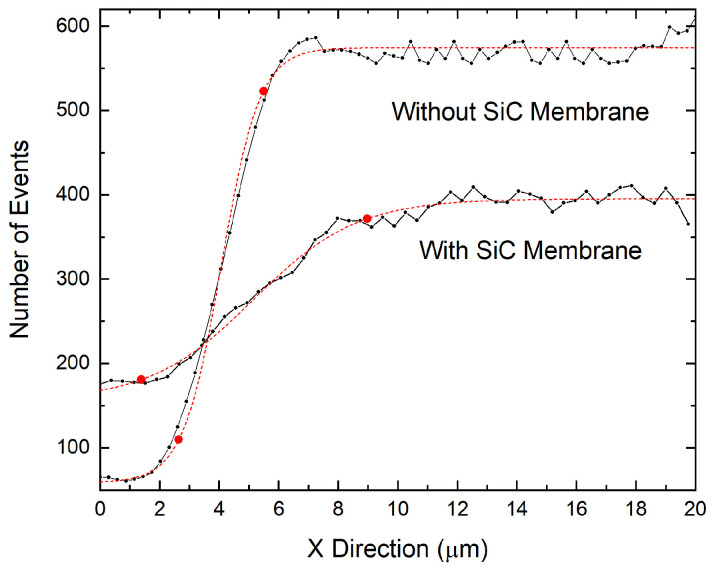
Count event profile close to the grid projection edges measured by the STIM detector without and with the SiC membrane sensor. The black dotted line represents the number of events measured as a function of position. The dashed red curves represent the Boltzmann sigmoidal function fitted to the experimental data. The points marked in red enclose the data interval between 10% and 90% of the upper plateau value.

The ion beam’s spatial profile in the two configurations was calculated using the knife-edge calculation procedure. Data from the regions near the grid edge shadow in the STIM map have been reported as the number of events versus position (Figure 4). The sigmoidal profile of the recorded events corresponds to the lateral profile of the scanning beam spot and was analyzed using a Boltzmann sigmoid function (the dashed red curves in Figure 4). The beam’s spatial profile was determined in the two scenarios (with and without the SiC free-standing membrane). This determination was carried out by calculating the number of pixels (Δx) falling in the range defined by the two points marked in red in Figure 4, which corresponded to the position of the 10% and the 90% values of the upper plateau of the function. Using this procedure, different measurements were carried out, and a main value of Δx = 11.84 ± 1.85 px was determined for the without-membrane configuration. The beam spot size could be calculated by multiplying the Δx main value and the pixel-to-micron conversion factor for this configuration (F_1_ = 0.29 µm × px^−1^), resulting in a beam spot size of r_Beam_ = 3.43 ± 0.54 µm. The same procedure was employed for the STIM data acquired with the beam passing through the SiC membrane sensor. With this configuration, a main value of Δx = 21.46 ± 1.74 px was obtained. The beam size after membrane interaction was calculated by considering the conversion factor F_2_ = 0.38 µm × px^−1^, leading to a beam dimension of D_Beam_ = 8.15 ± 0.66 µm. The high 8% error is ascribable to the roughness of the SiC membrane. The difference in the conversion factors F_1_ and F_2_ derive from the different magnifications at which the two STIM data were acquired. This difference is visible by comparing Figure 3b,c.

**Figure 5:**
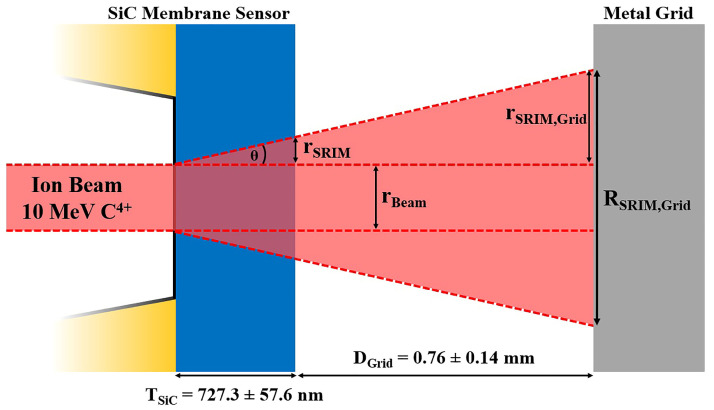
Geometrical representation of the ion beam path for theoretical lateral straggling calculation.

To verify the reliability of the experimental results, a simulation was conducted using the SRIM simulation tool to determine ion beam straggling under ideal conditions. Considering the experimental setup as well as the beam and sample characteristics, SRIM simulations offer precise calculations for various parameters, including but not limited to the ion range, implanted ion spreading (both transversal and longitudinal), and lateral straggling of transmitted ions. The latter parameter was employed to calculate the beam’s spatial profile, enabling the comparison with the experimental results. The lateral straggling parameter, as determined by SRIM simulations, characterized the beam’s lateral profile measured immediately after ions passed through the membrane, resulting in r_SRIM_ = 2.42 ± 0.30 nm. In order to obtain a parameter to be compared with the experimental result, the scheme of the setup, shown in Figure 5, had to be taken into account.

The metal grid, which shadowed the ion beam for the experimental measurement, was positioned at a distance of D_Grid_ = 0.76 ± 0.14 mm from the SiC membrane. It was essential to account for the beam divergence in this region. The SRIM spreading parameter r_SRIM_ was used to calculate the straggling angle θ_SRIM_ through trigonometric considerations. Using this methodology, we calculated the straggling angle relative to the incident direction of the ions, which resulted in θ_SRIM_ = 0.19 ± 0.02°. By knowing θ_SRIM_ and the distance D_T_ = T_SiC_ + D_Grid_, where T_SIC_ = 727.3 ± 57.6 nm is the thickness of the SiC sensor calculated previously, the lateral straggling r_SRIM,Grid_ on the grid could be calculated. With these considerations, a lateral beam straggling of r_SRIM,Grid_ = 2.53 ± 0.60 µm was obtained. To determine the total ion beam lateral profile, the initial beam dimension (r_BEAM_ = 3.43 ± 0.54 µm) had to be added to the spreading already calculated, resulting in a final beam dimension of R_SRIM_ = 8.49 ± 0.81 µm.

The comparison between the theoretical ion beam’s final dimension calculated using SRIM simulations (R_SRIM_) and the experimental beam dimension (D_Beam_) shows that the calculation method used in this work is quite reliable and allows the prediction of the ion lateral straggling in various scenarios. It also demonstrates the importance of mounting the sample to be implanted as close as possible to the membrane sensor to minimize the adverse effects of ion straggling. In scenarios involving heavy MeV ions, these distances should be less than 100 µm. Based on these results, further upgrades to the setup are planned. These upgrades aim to enable the mounting of the targets as close as 10 µm behind the membrane sensor, significantly enhancing the accuracy of the impact position of transmitted ions with the target.

## 4. Discussion

In deterministic ion implantation, the exact counting of ions as well as its spatial precision represent an ongoing challenge. The device presented here utilizes a membrane solid-state sensor and a low-noise, charge-sensitive electronic chain. This system collects a signal generated by ions transmitted through the sensitive membrane volume. In our experiments, the energy loss in the membrane active layer was about 1.5 MeV, and the number of generated pairs was on the order of 10^5^ pairs per ion. However, typical ion-implantation energies are in the range of a few hundred keV, which requires low energy loss inside the membrane and the detection of a signal derived by 10^3^ ÷ 10^4^ electron–hole pairs. These limits impose the use of a nanometric-thin membrane and a very low noise generated both by the detector and by the stage electronics.

Concerning the thickness of the sensor, a 100 nm SiC free-standing membrane can be produced using the ECE process described earlier. Thin membranes of this nature have already demonstrated favorable mechanical properties, including a high fracture strength and deformation [28]. The energy loss in ionization, i.e., the energy that ions lose in collisions with atomic electrons generating free charges in the solid material, depends on the mass and energy of the implanted ion. Using a SiC sensor with a 100 nm SiC epitaxy sandwiched between 20 nm and 70 nm aluminum electrodes, a typical dopant such as P at 250 keV loses a total energy of 190 keV in the sensor and generates approximately 9 × 10^3^ electron–hole pairs in the sensor active layer. With low noise, this system will allow the implantation of a deterministic number of 60 keV P ions. The detector noise is mainly determined by the leakage current and by the detector capacitance. The leakage current in our detector was sufficiently low (a few pA) thanks to the wide bandgap of the silicon carbide semiconductor. The capacitance, on the other hand, considerably influenced the noise level due to the large sensor area (~1.6 mm^2^) and the low sensor thickness (~730 nm). Although a 100-nanometer thickness may have a negative impact on the sensor capacitance, this effect can be substantially alleviated by reducing the surface area of the sensor, resulting in an enhanced signal-to-noise ratio. To minimize electronic noise, a custom charge-sensitive amplifier with effective capacitance matching of the input stage to the detector capacitance can be employed. Further experiments are in the planning stage to utilize an even thinner device along with upgrades to the signal-processing electronics.

Concerning the measured lateral ion straggling, the obtained value of 8.15 µm seems to be relatively high for single-ion-implantation applications. This high value introduces a significant level of uncertainty in determining the final position of the ions, potentially compromising the deterministic nature of the implantation process. However, it is important to note that the primary contribution to the final ion beam size is attributable to the divergence of the beam in the region between the SiC membrane sensor and the sample. In this work, this distance is represented by the distance between the SiC sensor and the metal grid (D_Grid_ = 0.76 ± 0.14 mm). The membrane straggling contribution was very low (r_SRIM_ = 2.42 ± 0.30 nm calculated with SRIM) compared to the final beam dimension. Therefore, by reducing the distance between the SiC sensor and the implanted sample, a higher determination of the ion’s final position can be achieved. For example, by reducing the D_Grid_ distance to a few micrometers (5 ÷ 10 µm), the membrane straggling contribution on the sample will be 19.1 ÷ 35.7 nm (calculated with the same θ_SRIM_ angle). In this case, the initial beam dimension r_Beam_ will strongly affect the final beam lateral profile (in this experiment, r_Beam_ was 3.43 ± 0.54 µm).

Hence, through the reduction in the initial beam size to a few tens of nanometers and the detector thickness to 100 nm, it becomes possible to attain a final beam size of approximately 100 nanometers. This reduction significantly mitigates the uncertainty associated with the final position of the implanted atom.

## 5. Conclusions

In this study, we introduced an innovative approach to single-ion detection during the ion-implantation process by utilizing an advanced SiC ultra-thin solid-state sensor. The sub-micrometer membrane enables ion detection through the creation of electron–hole pairs due to interactions between ions and the solid sensor without causing substantial interference with the ion trajectory, thereby enabling simultaneous control of the implantation process. The ion-beam-induced charge signal was collected for ions transmitted through the membrane and fully stopped in the PIPS detector positioned behind, resulting in a 96.5 ± 0.9% calculated ion counting confidence for the membrane. While this result can be improved by minimizing the signal background, it demonstrates the potential of utilizing a thin sub-micrometer membrane as a high-fidelity in-beam ion detector. Such a detector could be useful for novel true maskless deterministic implantation schemes needed for the fabrication of novel solid-state technologies and devices. However, the presence of the membrane interfered with the ion beam trajectory, introducing ion straggling effects that resulted in an increase in the uncertainty of the final position of the ion in the target. To quantify the ion straggling caused by the SiC membrane, a knife-edge measurement technique was employed, and the results were compared to SRIM simulations, yielding a final beam size of 8.15 µm. The observed high straggling value can be reduced through improvements in experimental conditions. These optimizations include minimizing the separation distance between the SiC sensor and the implanted sample to a few micrometers while simultaneously reducing the initial dimension of the ion beam to the nanometer scale and the membrane thickness to 100 nm. Implementing these conditions makes it possible to achieve a final beam dimension on the order of 100 nanometers, effectively minimizing the uncertainty associated with the final position of the implanted atom.

## Data Availability

Data are contained within the article.
